# Opportunities and limitations: A comparative analysis of citizen science and expert recordings for bioacoustic research

**DOI:** 10.1371/journal.pone.0253763

**Published:** 2021-06-28

**Authors:** Denise Jäckel, Kim G. Mortega, Ulrike Sturm, Ulrich Brockmeyer, Omid Khorramshahi, Silke L. Voigt-Heucke

**Affiliations:** 1 Museum für Naturkunde Berlin, Leibniz Institute for Evolution and Biodiversity Science, Berlin, Germany; 2 Life Sciences Faculty, Humboldt-Universität zu Berlin, Berlin, Germany; 3 Animal Behaviour, Institute of Biology, Freie Universität Berlin, Berlin, Germany; Texas Christian University, UNITED STATES

## Abstract

Citizen science is an approach that has become increasingly popular in recent years. Despite this growing popularity, there still is widespread scepticism in the academic world about the validity and quality of data from citizen science projects. And although there might be great potential, citizen science is a rarely used approach in the field of bioacoustics. To better understand the possibilities, but also the limitations, we here evaluated data generated in a citizen science project on nightingale song as a case study. We analysed the quantity and quality of song recordings made in a non-standardized way with a smartphone app by citizen scientists and the standardized recordings made with professional equipment by academic researchers. We made comparisons between the recordings of the two approaches and among the user types of the app to gain insights into the temporal recording patterns, the quantity and quality of the data. To compare the deviation of the acoustic parameters in the recordings with smartphones and professional devices from the original song recordings, we conducted a playback test. Our results showed that depending on the user group, citizen scientists produced many to a lot of recordings of valid quality for further bioacoustic research. Differences between the recordings provided by the citizen and the expert group were mainly caused by the technical quality of the devices used—and to a lesser extent by the citizen scientists themselves. Especially when differences in spectral parameters are to be investigated, our results demonstrate that the use of the same high-quality recording devices and calibrated external microphones would most likely improve data quality. We conclude that many bioacoustic research questions may be carried out with the recordings of citizen scientists. We want to encourage academic researchers to get more involved in participatory projects to harness the potential of citizen science—and to share scientific curiosity and discoveries more directly with society.

## Introduction

Citizen science (hereinafter abbreviated as CS) flourishes globally and has received significant recognition from diverse stakeholders in recent years. It is acknowledged for its potential to contribute to the transformation of the scientific system [1], promote global biodiversity monitoring [2], inform policies [3] as well as educate and promote scientific research in society [4]. In contrast to the traditional scientific research process, volunteers are involved in various activities of knowledge production for science and society [5]. CS is not a new hype: it has, especially in ornithology, a long tradition. For example, as early as 1749, one of the first CS projects in Finland collected data on migratory birds [6]. Today volunteers contribute large amounts of data in ornithological monitoring [7], which provide invaluable data for identifying trends in population numbers over time [8]. With the expansion of the internet and the increasing availability of user-friendly, cost-effective technology, citizen scientists got access to sophisticated data collection and transmission technology [9, 10]. Smartphone-based applications (mobile apps) allow citizen scientists to easily send photos, video, audio recordings, observation data and GPS positions [11]. This opened up new opportunities for CS in the field of bioacoustics, which otherwise depended on expensive equipment. In the field of ornithology, songbird dialects have been studied for decades [e.g. 12–14], with as well as without citizen scientists. So far, there are only a few CS projects with a focus on geographic variation in birdsong. One prominent European example is the Yellowhammer, *Emberiza citrinella*, with a detailed large-scale mapping of geographic variation of song dialects based on acoustic data collected by citizen scientists [14, 15]. Recently, a CS project based in North America successfully investigated the variation in chipping sparrow’s song [16].

Although CS data are increasingly recognized as both a complement to and a replacement for conventional data sources [17, 18], there is still an ongoing intense debate about challenges such as data quality [e.g. 19]. Both the lack of knowledge, skills and motivation of the participants [20, 21] and insufficient study design of CS projects [22, 23] are discussed as potential reasons for poor data quality. Interestingly, some studies have however shown that citizen scientists were more careful in their measurements and annotations because they were quite aware of their novice status [e.g. 24, 25]. Other studies have found a learning effect among citizen scientists and an increase in data quality over time [e.g. 26]. Nevertheless, this has led some scientists to generalise and thus, to *per se* consider CS data to be inferior to expert data (hereinafter abbreviated as EX; [25, 27]). At times, studies using CS data faced problems in being published in peer-reviewed journals [28]. Data quality, however, is a multidimensional measurement of accuracy, completeness, consistency and timeliness [29] that consists of a variety of attributes [30]. How data quality can be assessed strongly depends on the research question and thus on the parameters under consideration [for an overview see 31 or 32]. Comparisons between CS and EX data often focus on ecological aspects, i.e. the quality of species distribution maps [e.g. 33] or the occurrence of species for monitoring data [34]. In most cases, experts provided better monitoring data, because citizen scientists underrepresented [35] or overrepresented the species to be studied [36, 37]. Bernard and colleagues [38] found that monitoring data do not differ between citizen scientists and experts when frequent species with high detection probabilities were investigated. However, other studies have shown that—regardless of the frequency of occurrence of the species under investigation—citizen scientists produce equivalent data to experts, which were considered reliable and comparable [e.g. 26, 39, 40]. In this comparison, however, it is important to note that there are projects in which the knowledge, skills and accuracy of the citizen scientist are crucial for the validity and quality of data (e.g. eBird https://ebird.org/home). Additionally, there are projects in which the knowledge and skills of the citizen scientists are less important for the quality of the collected data, as these are generated for example with an app and subsequently checked by scientists (e.g. BirdNET [41], our study).

At present, studies on data quality of CS recordings in the field of bioacoustics, for example for song dialect research, are missing. To conduct dialect research, in particular, a large dataset with many recordings from many different males and regions is important as well as a high number of included songs and song types within the recordings. Especially in the field of song dialects, where the regional song variations between populations are studied over geographical distances, there is a great potential to use the power of CS. A high recording quality is required to be able to examine spectrograms, a visual way of representing the signal over time at various frequencies. For the investigation of regional variations, mainly the occurrence of song types is considered [40]. This song type classification can be performed semi-automatically by using cross-correlation or visual inspection of the recordings, which requires a high signal-to-noise ratio. Both approaches have already been successfully conducted in the song analysis of the common nightingale, *Luscinia megarhynchos* [42]. In nightingale song research, mainly nocturnal recordings have been used as these are easier to generate due to the continuous singing of the males at night. Further, the nocturnal song is more diverse due to its function of attracting females than the diurnal song, which males use for territorial defence [43]. There is yet no indication that certain song types are sung merely at night or during the day (personal observation). Nocturnal singing is also easier for humans to hear because of the largely low or absent background noise. The resulting higher recording quality also makes the nocturnal song more suitable for semi-automatic cross-correlation measurements.

**Fig 1:**
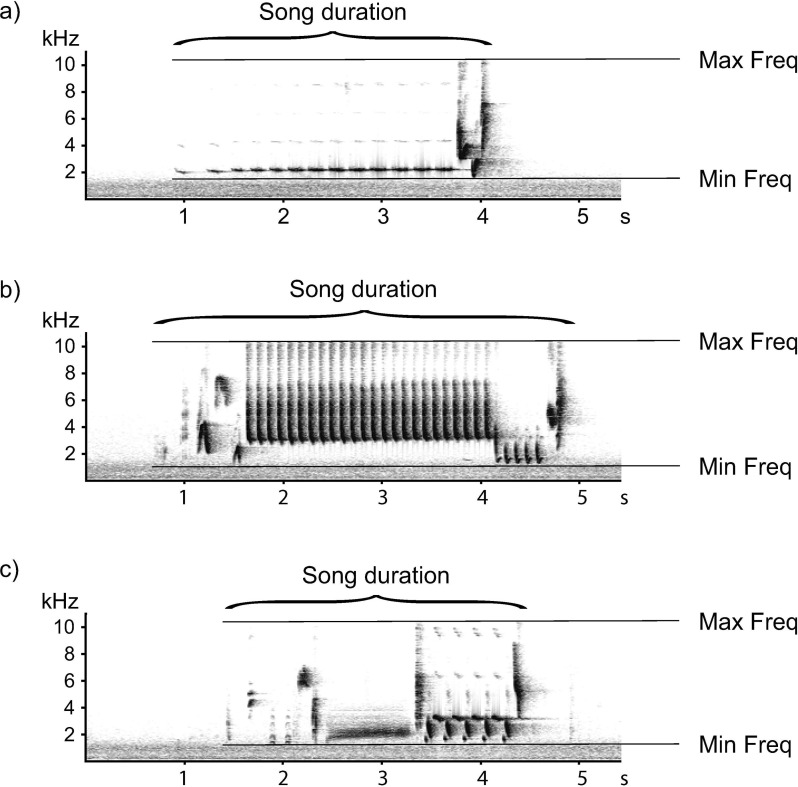
Exemplary spectrograms demonstrating the measured temporal and spectral parameters of the song duration, maximum frequency (Max Freq) and minimum frequency (Min Freq) in nightingale song for a) whistle songs, b) trill songs and c) buzz songs.

Nightingale song consists of several song categories which have, due to different volumes and spectral characteristics, different range characteristics and thus different signal-to-noise ratios. Whistle songs (Fig 1A) for example, have a long-range transmission [44, 45] whereas rapid trills (Fig 1B) degrade quickly over distance [45, 46], which means that their usability for semi-automatic cross-correlation measurements might be different. Thus, to better understand the impact of CS and EX recording devices on the recording quality, all song categories need to be tested. In addition, measurements of frequencies [e.g. 47] and durations [e.g. 48] have already been used in dialect studies with other bird species (MacGillivray’s Warbler, *Geothlypis tolmiei*, and grey-breasted wood-wren, *Henicorhina leucophrys*), although these have not yet been examined in CS recordings to assess the quality. Moreover, it has not yet been systematically investigated whether the assumption is valid that the use of different recording devices in the analysis of nightingale songs can be neglected due to their stereotypical song learning [49].

To contribute to the further development of CS in bioacoustics, we here compared the quality of nightingale song recordings collected either via a smartphone app by citizen scientists or with professional recording devices by EX in a case study. In the nightingale CS project, all citizen scientists were called upon to participate without restriction through various public channels (radio, newspaper, etc.). They did not receive any detailed briefing or protocols before or during the breeding season, nor did they receive any information or feedback for the exact generation of the recordings (time, place, duration, orientation of the smartphone, etc.). It can be assumed that due to the German species name "Nachtigall" (nightingale), which contains the word "Nacht" (night), many participants thought that the nightingale sings mainly or only at night. Furthermore, the nightingale is better heard at night due to the low (a)biotic and anthropogenic background noises, signifying to citizen scientist that nightingale males only or at least mainly sing at night. Midnight excursions offered as part of the project between 23:00 and 1:00 hours might have further confirmed these assumptions. In addition, we did not specify to the CS when in the breeding season they should generate recordings since, in the case of the nightingale changes in the breeding season such as declining song performance [50], lower number of different song types and higher repetition rate of the same song type (personal observation) have been found. This is why continuous recordings over the breeding season are also important for dialect studies in the nightingale.

We were particularly interested in the question of whether app recordings collected by citizen scientists are valid for identifying dialects in nightingales. To inquire this, we first examined the timing, quantity and quality of CS and EX recordings and then compared standard parameters in recordings generated simultaneously under identical conditions with either professional or mobile recording devices. We hypothesised that CS recordings made with smartphones are by large as valid for dialect studies in the nightingale as EX recordings. We predicted that 1) the CS recordings differ in the temporal coverage to EX recordings (H1: time of day/calendar week), 2) the CS recordings are of comparable quantity and quality to the EX recordings (H2: data quality), 3) the CS recordings are more likely to be valid with an increasing number of individual recordings (H3: improvement) and 4) the CS and EX recordings differ in recording quality because of the technical differences of the devices (H4: microphone comparison).

## Material and methods

### The nightingale as model species

The common nightingale (*Luscinia megarhynchos*) is a well-suited model species for a CS project in Berlin, as it is omnipresent in spring from around mid-April to late June during day and night. Its charismatic song is easy to identify even for laypeople and recordings of nocturnal songs easily reach a good recording quality, as there is hardly any background noise or other bird species to be heard. In addition, the song of the nightingale is so loud (74 dB (A) at 1 m distance [51]) that it is easily perceived by humans even from a distance and can thus be well recorded. Males possess an extraordinary large song-type repertoire (approx. 180 different song types per male) and differ considerably in repertoire size [49]. The song is mostly examined and classified on the song type level. Noteworthy categories of songs include whistles, trills and buzzes (Fig 1). Due to its highly complex song, the nightingale is also an interesting prospect model species for dialect studies. However, this research question has not yet been investigated for this species.

### Citizen science recordings—the ‘nightingale citizen science project’

We conducted the nightingale citizen science project at the Museum für Naturkunde Berlin (MfN), Germany. In spring, the project invited participants to generate nightingale audio recordings with their smartphone via the mobile app ’Naturblick’ (see more details [52]). The app’s pattern recognition supported citizen scientists in identifying audio recordings by presenting the top three candidates of each classification run [53, 54]. We designed the project with a very low threshold of participation in order to engage as many people as possible. The CS project therefore only set the target to record singing nightingales with the app. We did not provide any explicit specifications as to where, when, how long and how often to record a nightingale song. The ’Naturblick’ app also encouraged users to share a nightingale recording with the CS project if it was taken by chance. The maximum duration of the recordings was limited to two minutes due to technical reasons. The citizen scientists could decide for themselves whether they wanted to share their recordings anonymously or with an individual username. The project was conducted over a two year period (2018–2019). Since twice as many nightingale song recordings were generated in the second project year, we used only the CS data from 2019 from Berlin for all further analyses. All CS data was based on recordings with the smartphone app ’Naturblick’ (sampling rate = 44,1 kHz, bitrate: 256 Kbit/s, audio encoder: AAC Low Complexity (AAC-LC) audio codec). GPS coordinates were automatically included in the metadata for all recordings.

### Expert recordings

For the EX recordings, we used different datasets, which were created in the same time period as the CS data with expert equipment by academic researchers. First, students of the Freie Universität Berlin (FU) recorded nine nightingale males as part of a master course in four locations of Berlin ‘Volkspark Friedrichshain’ (52°31’39.9648”, 13°25’58.656”), ‘Dreipfuhl’ (52°26’49.272”, 13°16’19.6752”), ‘Rehberge’ (52°35’7.7244”, 13°11’6.1512”) and ‘Tiergarten’ (52°30’51.0804”, 13°21’38.3076”) between 22 May and 04 June 2018. The FU data were recorded using a Sennheiser microphone (Sennheiser ME66/K6 directional microphones; 44,100 Hz, 16-bit resolution) connected to a Tascam Dr-40 4-Track Portable Digital Recorder. Second, twelve additional one-hour long nightingale recordings were generated by academic experts of the MfN between 28 April and 07 May 2019 in Berlin. We recorded spontaneous nocturnal songs for individual, non-banded males in the field during the established recording time between 23:00 and 2:00 hours. We recorded three males at about the same time at night in the same area. This resulted in six recordings in the communal park ‘Volkspark Friedrichshain’ (52°31’39.9648”, 13°25’58.656”) and six recordings in a green space in the area of ‘Altglienicke’ (52°24’27.524”, 13°31’3.1476”). We used three professional recording devices (two Zoom H2n recorder and a Marantz solid-state recorder PMD660 (sampling frequency: 44,1 kHz; resolution: 16 bit) with a Sennheiser ME66/K6 Microphone (Georgsmarienhütte, Germany). The microphones were equipped with windbreakers. For later analyses, we randomly selected six recordings from 2018 and six recordings from 2019. Here we did not aim for a comparison between the years, but we rather aimed to use a wide selection of different EX recordings.

### Verification of recordings

All audio recordings were visualised for further analyses using Avisoft SASLab Pro 5.2 (R. Specht, Berlin, Germany). As the recordings via the app were generated in MP3 and m4a formats the recordings were transferred into the WAV format to be opened by Avisoft. For this purpose, we used the program WaveLab 7. Audio analyses were conducted using the same settings (sampling rate = 22,050 Hz, FFT = 1024 points, Hamming-Window, overlap 93,75%). The CS recordings were analysed visually and acoustically for nightingale songs, nightingale calls, the song of another bird species but which was not a nightingale, and no birds. A very small number of well-trained citizen scientists (n = 4) supported this step of recording classification. We only used nightingale songs for further analysis.

### Comparison between the recording times of the CS and EX group

We determined the time of day and calendar weeks for all recordings. As recommended in the literature [49], the EX recordings were made at standardised times (between 23:00–3:00 hours) when nightingales are particularly reliably singing—especially in the beginning of the season—and the SNRs are most likely high due to the low background noise. The citizen scientists, on the other hand, had no instructions as to when they should record. Since the probability of making many and valid recordings on the one hand at night and on the other hand at the beginning of the season is high, we assumed that the times for recording CS and EX would therefore overlap. The time and date of the CS recordings were recorded directly via the ’Naturblick’ app, actively shared with the CS project and delivered as metadata.

### Comparison between the relative percentage of valid recordings in CS and EX data

For the comparison between approaches, we used the CS recordings from 2019 (n = 5679) and the EX recordings from 2018 (n = 6) and 2019 (n = 6). We evaluated the relative percentage of recordings of nightingale song, other bird species and no birds (all recordings = 100%; number of real nightingales / 100% = relative percentage). Furthermore, we categorized the nightingale song recordings as to whether at least one song type in its entirety was recognizable by both, syllables and elements in the spectrograms (in the following abbreviated as ‘ist’ = identifiable song types) or to a lesser degree, i.e. some syllables or elements were not clearly shown in the spectrograms (‘nist’ = non-identifiable song types). The former were seen as indicators of a valid recording quality, the latter of a reasonable recording quality that could however not be used for dialect research based on the identification of song types. We examined the cumulative duration of recordings in order to determine the scope of the dataset. The duration of the CS recordings was supplied directly by means of the metadata. This includes the entire duration of the recording, but not the start and end of singing within the recording.

### Comparison between the relative percentage of valid recordings in CS data among different user types

Based on their username and the number of recordings that they shared with the project, citizen scientists were divided into three user groups: 1) one-time users who had generated only one recording (one recording), 2) frequent users who made several recordings (two to nineteen recordings) and 3) power users who made many recordings (more than 19 recordings). This classification was based on the graphical distribution of the number of recorders and the number of recordings. This curve flattened out at 20 recordings per participant. For the quantitative analysis, we used the parameters described above. Furthermore, we examined the number of songs within a recording, since a recording’s duration does not indicate how many songs are included.

### Comparison between the signal-to-noise ratio (SNR) in CS and EX recordings

We examined all song categories for potential differences, as the nightingale’s song categories have different transmission characteristics and thus different signal strengths. We selected three different song types from each song category (three whistles, three buzzes, three trill songs = in total nine different song types) for ’ist’ CS nocturnal recordings from 2019 and the EX recordings from 2018 and 2019. For each of these song types, we randomly selected a sample of 10 recordings out of each data source from the Berlin ‘Volkspark Friedrichshain’. We used the R-package warbleR [55] to automatically determine the signal-to-noise ratios (SNR) of recordings. For this purpose, the start and the end of a song were selected via an interactive spectrogram display in R using the mouse cursor. The SNR values were then automatically determined for the marked area. Referring to Araya-Salas and colleagues [56], we defined recordings to be of valid quality if they had a SNR over 10 dB. However, other authors recommend lower thresholds for the SNRs, such as Barmatz and colleagues [57, 58].

**Fig 2:**
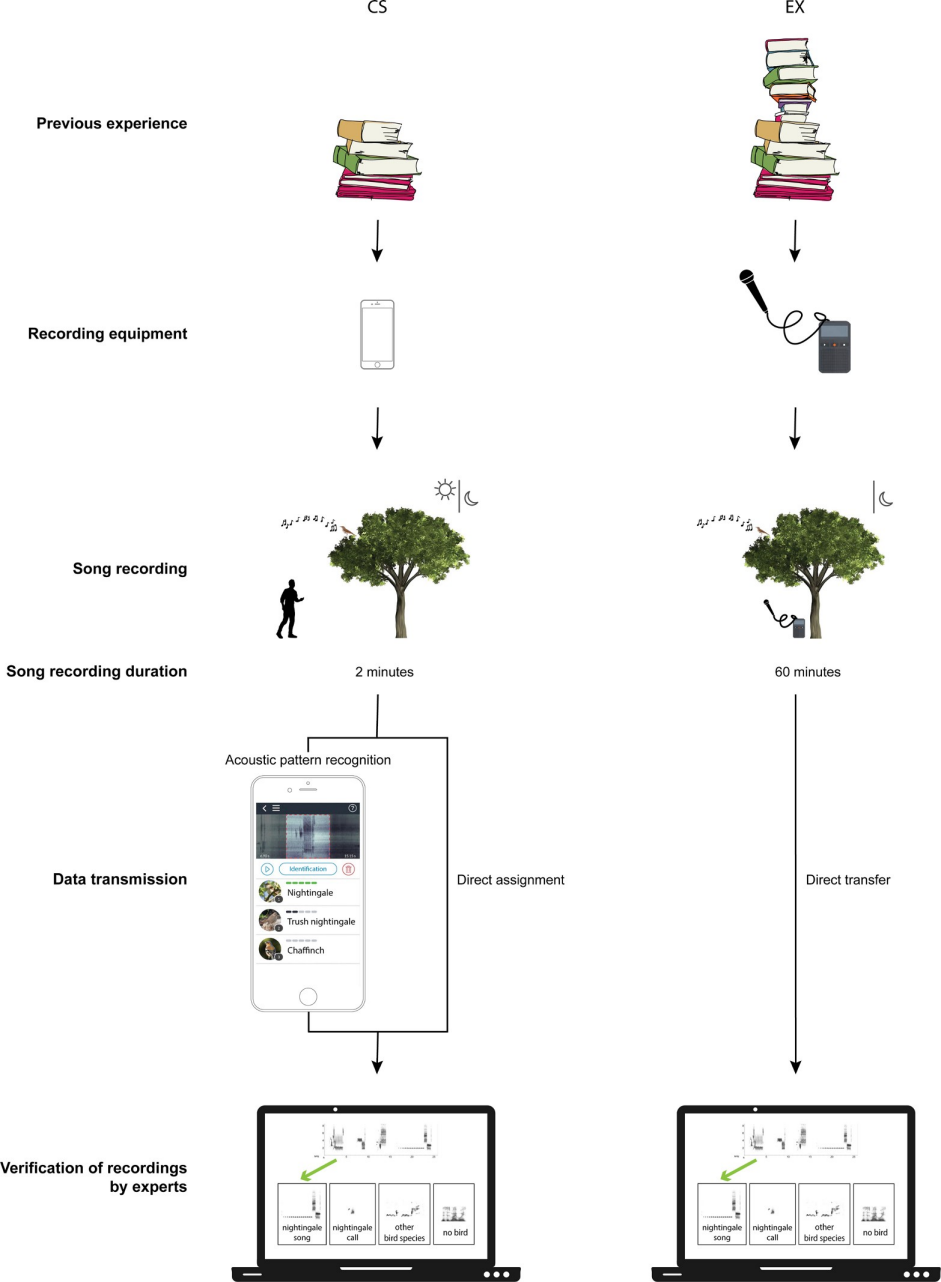
Process of generating recordings of CS (left) and EX (right). The similarities and differences in previous experience, recording equipment, song recording, song recording duration, data transfer and verification of recordings by experts are pointed out (from top to bottom).

For both CS and EX recordings, we lack information about exact distances to the singing bird. We assumed that the citizen scientists approached singing males as closely as possible. The EX recordings were conducted by placing a professional recorder underneath a song post of a prospective male (see Fig 2). At night, nightingales hardly move but remain sitting on their song posts. During the day, males move around more often (personal observation); they are marking their territory by singing and therefore make use of several song posts located on the border of their territory. In previous studies, SNR values were also obtained without direct distance measurement to the bird [57, 58]. These studies evaluated the usability of monitoring recordings in terms of their SNR values. Likewise, we here aimed to evaluate via a SNR analysis whether the CS recordings were valid to determine song types by semi-automatic cross-correlation.

### Comparison between the playback test recordings of CS and EX recording devices (smartphones vs. professional equipment)

To test whether measurements of spectral and temporal parameters would be influenced depending on the very different devices used by citizens or experts, we performed a standardized playback test. In September 2020, we simulated a singing nightingale and recorded it with several devices that differed considerably in terms of both, recording quality and price. In this simulation, a loudspeaker (JBL Charge 3) was placed on a chair 8 m away from a bench on a windless and sunny day. The playback was done at 8 m spacing, as in our personal experience this is a good average for the natural distance in the field when recording a nightingale. On the bench, in total 12 different recording devices were placed side by side and the microphones of the smartphones were aligned facing the loudspeaker. The devices were positioned in a horizontal position, as this by our experience seemed to be the most common position by citizen scientists when recording with a smartphone.

We based the choice of smartphone brands used for the playback on those that were most frequently used for the CS app recordings. The sensitivity of the smartphones was not standardised via the gain settings. As expert equipment, we tested recording devices that have been used for generations for the EX recordings (Zoom H2n recorder and Marantz solid state recorder PMD660 with a Sennheiser ME66/K6 Microphone (Georgsmarienhütte, Germany)). As smartphones, we tested 10 different devices widely used: the smartphone brands Apple (three devices), Google (two devices), HTC (two devices) and Samsung (three devices). The loudspeaker was set to 74 dB (A) at a distance of 1 m using a calibration device (TEAC Df-1). This corresponds to the natural source level of a nightingale [51]. The loudspeaker was used to broadcast a recording that contained three whistles, buzz and trill song types each (duration of the audio file = 96 seconds). We chose different song categories and within these three different song types to cover different frequencies and to check the frequency response of the various devices. We did not perform a standardised test where different frequencies are independently assessed, as we intended to test the devices under natural conditions. To compare the recording quality of all devices and their built-in/external microphones, the audio was then simultaneously recorded: for the smartphones with the ’Naturblick’ app and in the case of the two recording devices on the built-in data carrier. As a standard, all recorders had two built-in microphones. By default, we used only the first channel of the recordings for our following measurements. Subsequently, we performed standard measurements of spectral properties of the song type in the spectrogram, i.e. the minimum and maximum frequency, as well as the duration of the song types, was measured (Fig 1). We defined the song type duration as the duration from the beginning of the first to the end of the last element (in seconds). The measurements of acoustic parameters (frequencies and song duration) were done for the original recordings, which were played back in the test, as well as for the generated recordings of the different devices. One person carried out measurements twice manually in Avisoft. The spectrogram settings were adjusted in advance. For the three parameters (minimum frequency, maximum frequency and duration) we then averaged the values obtained for all song types and categories, determined the deviation from the measured values of the original recording and compared the results among the devices.

### Statistical analysis

To test for differences in the quality (SNR) of recordings between the CS and EX approach, we used Welch tests for normally distributed data and Wilcoxon signed rank tests for not normally distributed data: We used Friedman tests to compare differences among parameters (minimum frequencies, maximum frequencies and durations) and subsequent Nemenyi-Wilcoxon-Wilcox tests as a post hoc test. We set statistical significance at p ≤ 0.05. All statistical analyses were performed using R (version 4.0.0).

### Ethics statement

This study compares the quantity and quality of CS and EX recordings of nightingale song. The data of the citizen scientists were shared with our project with their approval via the ’Naturblick’ app. The EX recordings were made during a university course at the ‘Freie Universität Berlin’. For both types of recordings, we obtained the consent of participants to analyse their data. In Germany, the approval of an ethics committee is not required for such research questions and was therefore not obtained. We have therefore received all the necessary permissions required in Germany.

## Results

In total, more than 3,000 citizen scientists recorded a cumulative 82 hours of song and 35,462 songs without exact specifications as to when and how often to collect data. The EX recordings contributed a cumulative 12 hours of song and 4,921 songs to the study’s dataset. The CS and EX recordings cannot be compared in terms of these overall figures, as they were recorded with different specifications: CS—no time specifications when, how and how long they recorded; recording limit of two minutes, and EX—time specifications when, how and how long they recorded; recording limit of one hour. For this reason, in all further comparisons, we used the relative percentage of valid quality recordings rather than the total number and focused on the quality of the data that could be used for further bioacoustic analysis.

### Recording times of citizen scientists and experts

**Fig 3:**
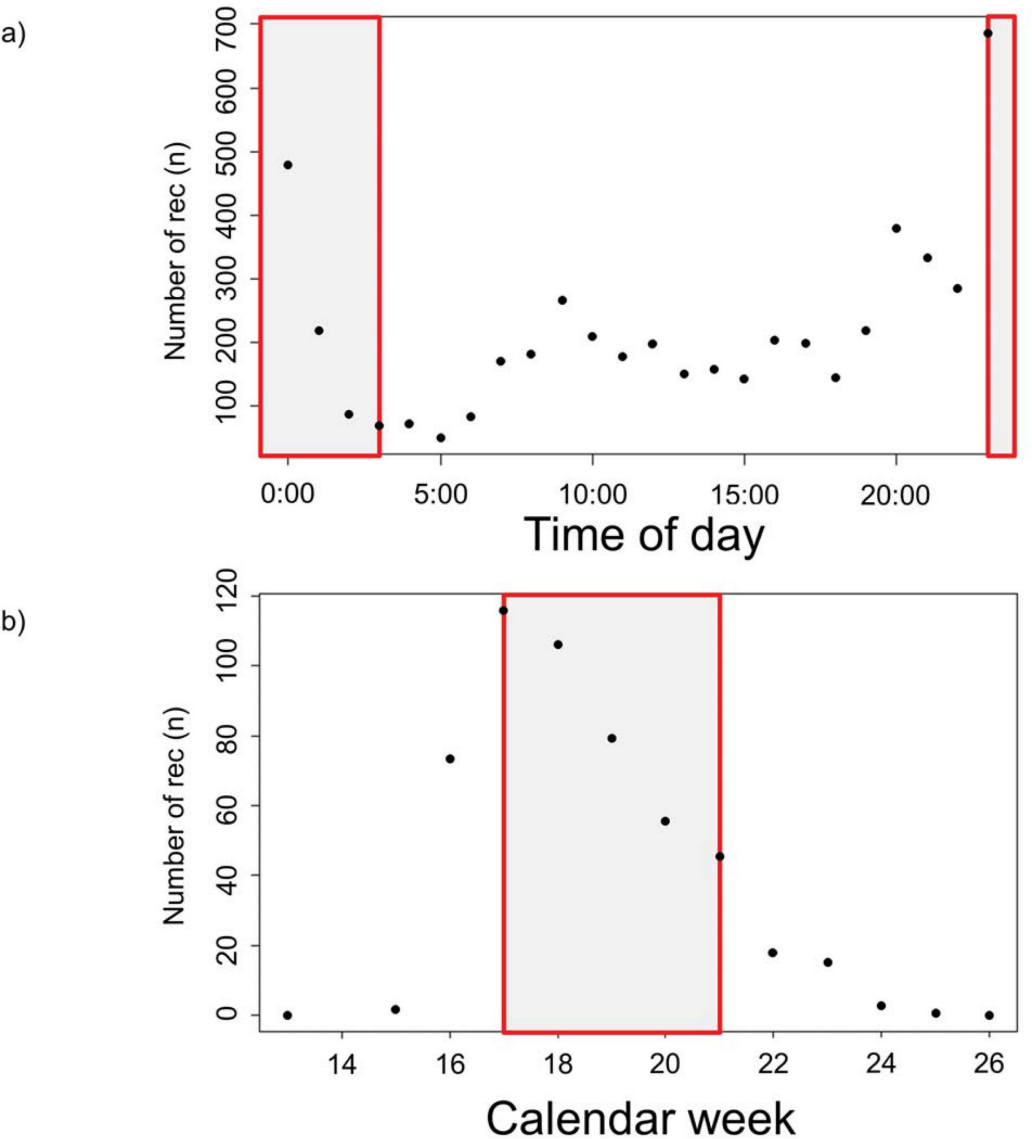
Temporal distributions of 5,679 citizen science recordings (abbreviated as rec) with the ’Naturblick’ app for 2019. In red, the times of previous six nightingale studies are shown (2004–2017; [61–64, 79, 80]). a) Representation of the number of recordings in relation to the time of day. b) Presentation of the weekly number of recordings in the course of the breeding season. https://doi.org/10.5281/zenodo.4817236.

Most CS and EX recordings were generated between 23:00 and 00:00 hours (Fig 3A). Overall, CS recordings were made during all times of day without any temporal gaps. The fewest CS recordings were made between 3:00 and 4:00 hours. In total, citizen scientists generated recordings between the 16th and the 26th calendar week. Most recordings were generated between the 17th and 21st calendar week by both, the CS and EX group (Fig 3B). The fewest CS recordings were made in the 13th, 25th and 26th calendar week.

### The relative percentage of recordings with valid quality in CS and EX data

**Fig 4:**
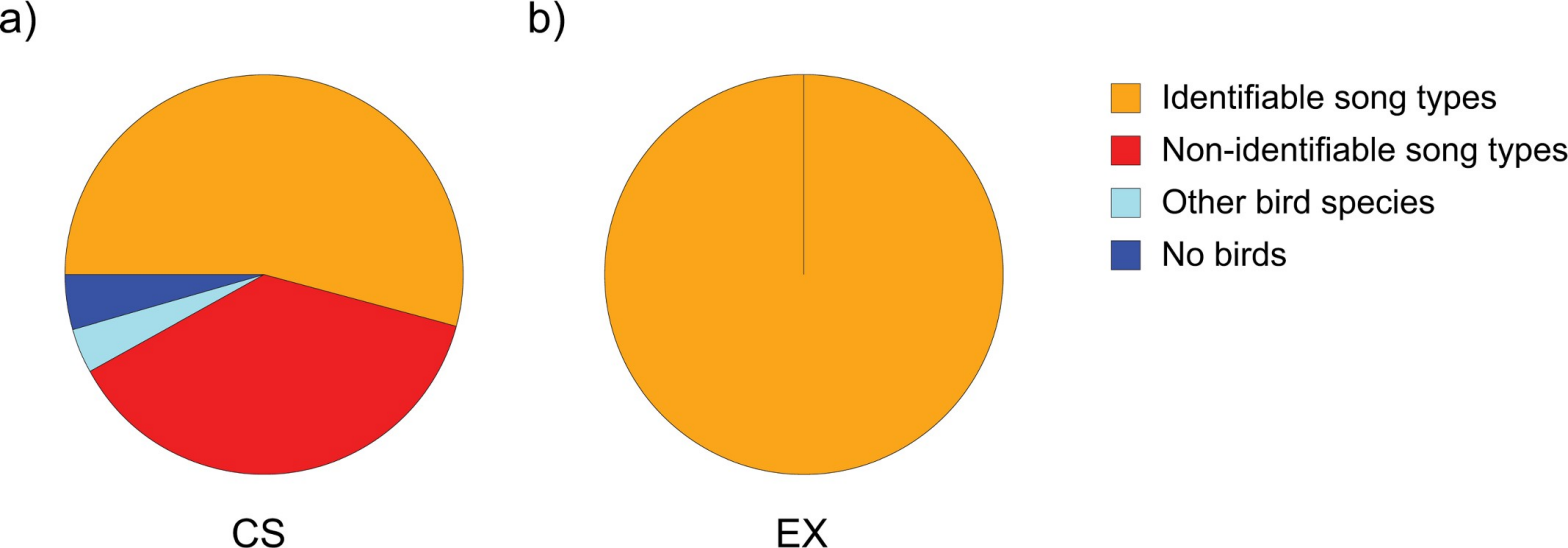
Comparison of defined recording categories for 2019, based on a) 5,679 citizen science recordings (CS) with smartphones via the ’Naturblick’ app and b) six expert recordings (EX) with high-quality microphones. The categories are displayed in different colours (red: one song type was in its entirety recognizable by syllables and elements in the spectrograms, orange: some syllables or elements were not clearly shown in the spectrograms, light blue: other bird species and dark blue: no birds). https://doi.org/10.5281/zenodo.4817236.

The comparison between the CS and EX recordings showed that the EX group produced 100% ’ist’ recordings and the CS group 53% of ’ist’ recordings (S1 Table). In addition, nightingale recordings were sent from twelve countries within the CS project. The EX recordings come from one country. The CS group also generated 37% ’nist’ recordings, 2% call recordings, 4% other bird species and 4% ‘no bird‘ recordings (for 2019 see Fig 4). The mean duration of the recordings was higher for the EX recordings (60 minutes) than for the CS recordings (54 seconds). The cumulative recording time was higher for CS recordings (89 hours) than for EX recordings (6 hours).

### The relative percentage of CS recordings with valid quality for single, frequent and power users

**Fig 5:**
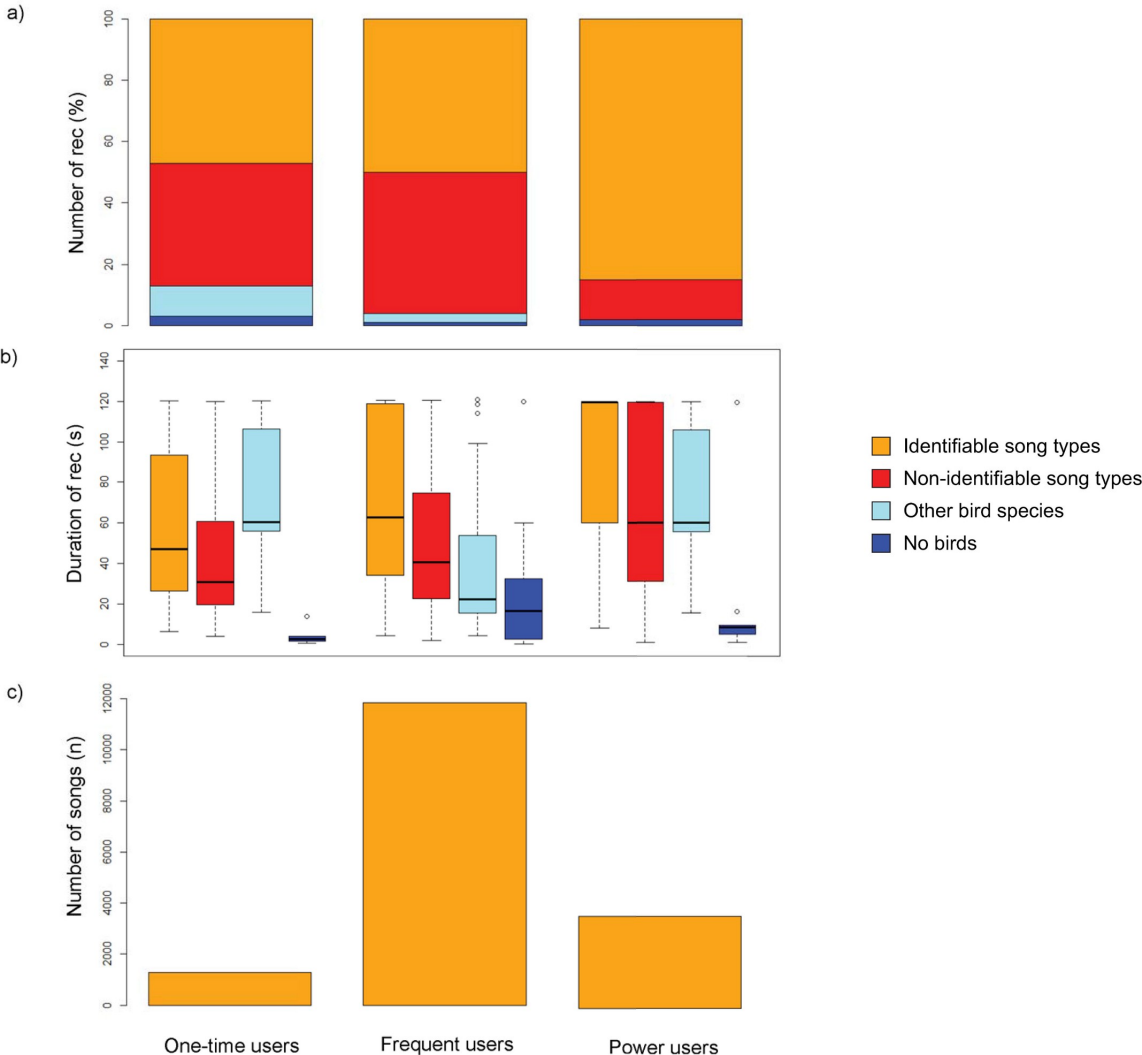
Comparison of the number of citizen science recordings (rec) in the year 2019, based on recordings with the ’Naturblick’ app of 245 one-time users, 361 frequent users and 18 power users (one-time users = 1 recording; multiple users = 2–19 recordings; frequent users ≥ 20 recordings). In red: one song type was in its entirety to be identified by syllables and elements in the spectrograms, in orange: some syllables or elements were not clearly shown in the spectrograms, in light blue: other bird species and in dark blue: no birds. a) The number of recordings. b) The duration of recordings shown in the boxplots. The median is represented by a solid black line and the mean by a dashed black line within a box. The borders of boxes are 25 and 75 percentiles. The bars above box plots indicate significant differences between two stimulus categories. c) Shows the cumulative number of songs in all user groups for the first category. https://doi.org/10.5281/zenodo.4817236.

The comparison among user types showed that the frequent users had the highest number of all categories of recordings, the second-longest average recording time and the longest cumulative recording time (S2 Table). Power users had the highest percentage with 85% of ‘ist’ recordings, whereas the percentage of single and frequent users was similar with 50% and 47% (Fig 5A). Conversely, power users generated the lowest percentage of ‘nist’ recordings (13%), followed by one-time users (40%) and frequent users (46%). Within all the user groups, ‘no bird’ song recordings made up the lowest percentage of recordings (power users = 2%, frequent users = 1%, one-time users = 3%). Power users had the longest mean duration of ‘ist’ recordings with 99 seconds, followed by frequent users with 72 seconds and single users with 59 seconds (Fig 5B). Among power users, the ‘nist’ recordings had nearly the same total duration (67 seconds) as the category ‘other bird song recordings’ (70 seconds). The ‘nist’ recordings were longer in their total duration than the total duration of ‘no bird‘ song recordings (6 seconds) for the power users, and this was the other way round for the single users (46 seconds to 5 seconds). For all user groups, the ‘no bird’ song recordings were the shortest (single users: 5 seconds, frequent users: 24 seconds, power users: 6 seconds). The cumulative number of songs differed between the user groups with frequent users having the largest cumulative number of songs (n = 11,845), followed by power users (n = 3,602) and single users (n = 1,288; Fig 5C).

### The signal-to-noise ratio (SNR) in CS and EX recordings

The EX recordings had in all song type categories a mean SNR that was higher than 10 dB. The CS recordings only had a median higher than 10 dB for the song type category whistle and trill. The SNRs of CS data differed significantly among all song type categories (whistle, trill and buzz) from the EX data (whistle: Wilcoxon signed rank test: W = 287, p-value = 0.016, trill: Wilcoxon signed rank test: W = 316, p-value = 0.048, buzz: Welch test: t = -5.5705, df = 56.704, p-value <0.001). The number of valid recordings determined by SNR values above 10 dB (according to Araya-Salas and colleagues [55]) was higher for EX recordings (in total—whistle: 23, trill: 27, buzz: 24) than for CS recordings (in total—whistle: 17, trill: 11, buzz: 18, Fig 6B).

**Fig 6:**
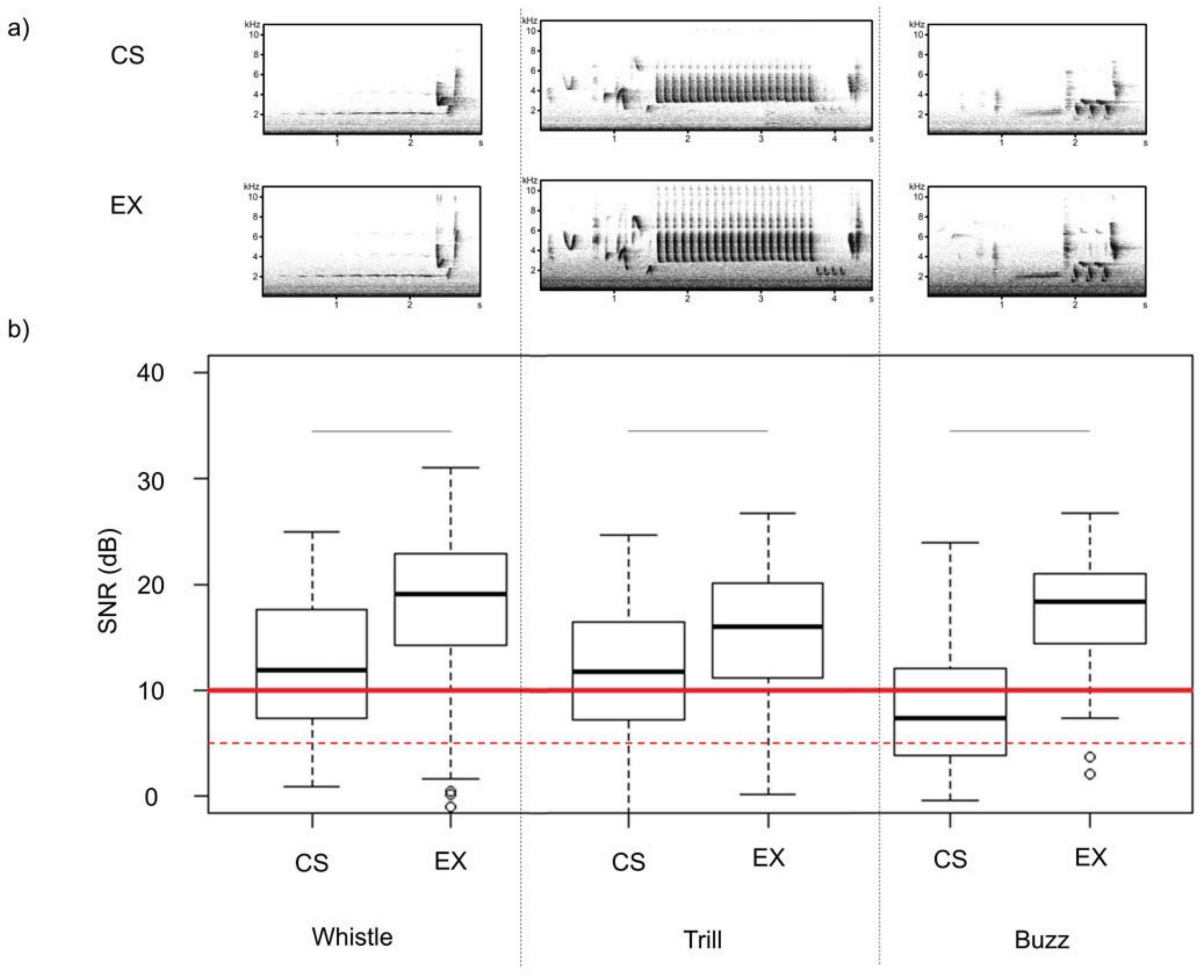
Comparison of citizen science (CS) recordings with the ’Naturblick’ app and expert recordings with equipment using professional microphones (EX) in the year 2018 and 2019. a) Spectrograms of CS recordings (top) compared to EX recordings (below) for the one example per song categories (whistle, trill and buzz). b) Comparison the signal to noise ratio (SNR) as boxplots for three of these selected song types per each category: whistle (left), trill (middle) and buzz (right). The median of the boxplot is represented by a solid black line and the mean by a dashed black line within a box. The borders of boxes are 25 and 75 percentiles. The bars above box plots indicate significant differences between two stimulus categories. SNRs over 10 dB (red line) were defined in this study as valid quality according to Fitzpatrick and colleagues [58]. The red dotted line indicates a value of 5 dB, a threshold used by Barmatz and colleagues [57, 58] forvalid quality. The black lines indicate significant differences. All recordings were examined and displayed under the same settings (sample rate = 22,050 Hz, FFT = 1024 points, Hamming-Window, overlap 93,75%). https://doi.org/10.5281/zenodo.4817236.

### Playback test for recordings of CS and EX recording devices (smartphones vs. professional equipment)

**Fig 7:**
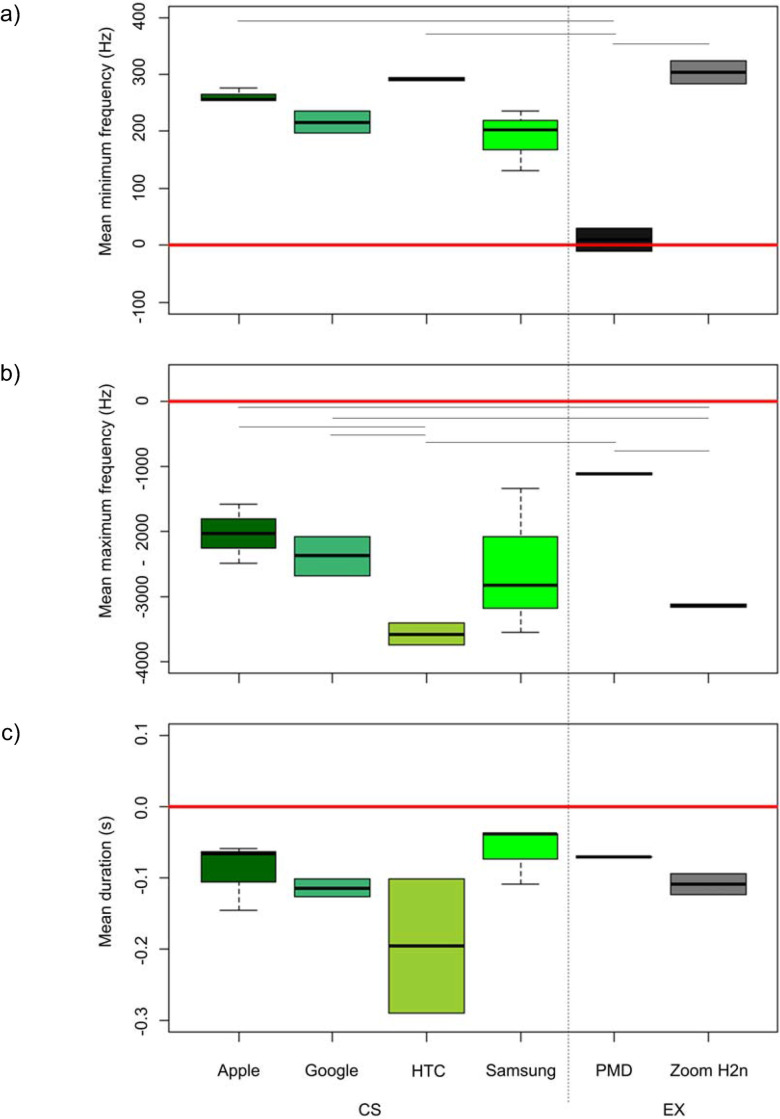
Comparison of deviations from original playback files of the different recording devices, i.e. smartphone brands (CS) and expert recordings equipment with professional microphones (EX) in a test. Depicted in boxplots is the deviation in a) the minimum frequency, b) the maximum frequency and c) song duration from playing back a test file of nightingale song. The median is represented by a solid black line within a box. The borders of boxes are 25 and 75 percentiles. The red line shows a zero line. The closer a deviation to the zero line is, the smaller it was. The black lines above boxplots indicate significant differences between the recording devices tested.

**Fig 8:**
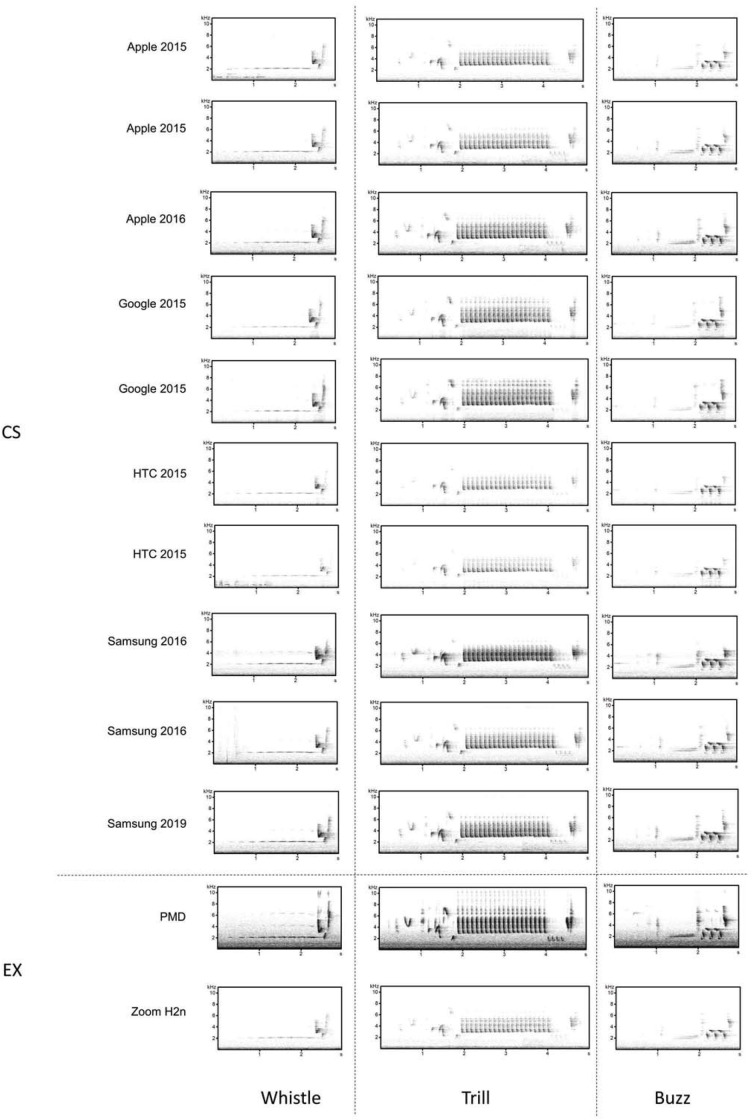
Comparison of citizen science (CS) recordings with the ’Naturblick’ app and expert recordings with equipment using professional microphones (EX) during a playback test. Spectrograms of CS recordings (top) compared to EX recordings (below) for the one example per song categories (whistle, trill and buzz).

We found significant differences between the recording quality of different recording devices, expressed by the deviation from the minimum and maximum frequency as well as duration from the original playback file (Figs 7A–7C and 8). Overall, the professional PMD recording device showed the lowest deviation from the original recording in the minimum and maximum frequency measurements, while the also professional Zoom H2n and the HTC smartphones showed the highest deviation. We detected significant differences among recording devices in the deviation from the minimum frequencies of the test playback file (Fig 7A; Friedman test: F = 26.079, n = 6, df = 5, p-value <0.001). We detected the greatest deviation from the original recording in the minimum frequency for the professional Zoom Hn2 and the smallest deviation was measured for the professional PMD recording device. Post-hoc test revealed that measurements for the professional PMD recording device differed significantly from the smartphone brands HTC (Nemenyi-Wilcoxon-Wilcox, n = 2, p = <0.001) and Apple (Nemenyi-Wilcoxon-Wilcox, n = 2, p = 0.004) as well as from the professional Zoom H2n (Nemenyi-Wilcoxon-Wilcox, n = 2 p = <0.001). We found significant differences in the deviation from the maximum frequencies of the test playback file among the recording devices (Fig 7B; Friedman test: F = 37.444, n = 6, df = 5, p-value <0.001). The smartphone brand HTC had the largest deviation at the maximum frequency from the original recording and the professional PMD had the smallest deviation. Post-hoc tests showed that the deviation of the maximum frequencies of the HTC smartphones differed significantly from the smartphone brands Google (Nemenyi-Wilcoxon-Wilcox, n = 2, p = 0.004) and Apple (Nemenyi-Wilcoxon-Wilcox,n = 2, p = 0.002) as well as the professional PMD recording device (Nemenyi-Wilcoxon-Wilcox, n = 2, p < 0.001). Moreover, post-hoc tests revealed that the deviations of the professional Zoom H2n differed significantly from the smartphone brands Google (Nemenyi-Wilcoxon-Wilcox, n = 2, p = 0.044) and Apple (Nemenyi-Wilcoxon-Wilcox, n = 2, p = 0.03) and the professional PMD recording device (Nemenyi-Wilcoxon-Wilcox, n = 2, p < 0.001). The recording devices did not differ among their deviations from the original duration of the playback file (Fig 7C; Friedman df = 5, n = 2, p-value = 0.063; for details see S3 Table).

## Discussion

This study highlights the potential of citizen science for bioacoustic research. We found that bioacoustic research—for instance here, dialect research on the nightingale—could be carried out both with the recordings of citizen scientists and experts. The frequently discussed lack in the overall data quality of CS data could not be confirmed in this case study. Instead, we were able to show that the quality of CS recordings was in large parts equivalent and not *per se* inferior to EX recordings. Furthermore, our study confirms the notion that CS has the advantage to generate large datasets. In the following, we discuss the two aspects that we believe may have influenced our results: the human and the technical factor.

### The human influence on CS data

Many studies comparing citizen scientists with experts assume that the latter *by proxy* generate better data due to their extensive experience and scientific background [59, 60]. However, in our study, the knowledge and skills of the citizen scientists were less decisive for the recording quality, as participants were able to validate their recordings with a pattern algorithm and data was additionally verified by experts. We infer from our results that previous experience is not always required when collecting data that is mainly relying on good technical equipment. Our comparison of the recording times between the two approaches (non-standardised CS times vs. standardised expert times) showed that citizens’ studies without a specification achieved a much higher temporal and spatial resolution than previous nightingale expert studies. Academic researchers are usually looking for valid quality as well as long recordings; thus, they usually specify research designs with standard recording times in which they can expect valid singing performances of their study objects. In consequence, this leads to a sometimes narrow temporal recording window and consequently, the bandwidth of recording times and possible variations in singing behaviour of study species may be lost. Earlier nightingale studies mainly used the nocturnal song for their analyses, as this signals an unpaired status of the males and allows a comparison between the males under study and their songs [e.g. 61]. Nevertheless, recordings outside the night (thus dawn and day) and over the whole breeding season are of crucial value for dialect research. Firstly, the number of different song types decreases over the breeding season and the repetition rate of the same song type increases (personal observation). And secondly, it has not yet been investigated whether the song types differ between night and day. The CS recordings covered not only the whole breeding season but the whole day. Most CS recordings were without specifications made during the same time (day and week) as previous nightingale studies [e.g. 49, 61, 62]. The majority of recordings for both approaches were made at night. We detected two recording peaks at 9 am and 8 pm. The first peak was probably created when most people were on their way to work and the second one when people actively went out to listen to nightingales in the beginning of the night. It is worth mentioning that there is no time of the day during which a song recording of a nightingale was not made in the course of the CS project. This indicates that the CS recordings have much greater temporal coverage because they were made throughout the day. Also, the weekly coverage by citizen scientists was greater than by the experts in earlier nightingale studies [e.g. 49, 61, 62].

Without having precise specification, citizen scientists generated the most recordings between the 17th and the 25th calendar week. Standardized recording times in previous nightingale studies were usually between the 17th and 18th calendar week [63], up to 21st calendar week [64] or up to 22nd calendar week [62]. Thus, without having any concrete guidelines, following the singing pattern of the nightingale resulted in similar and partly overlapping recording periods. The decreasing number of CS recordings as the breeding season progressed is certainly also due to the fact that with more male nightingales being paired, the nocturnal singing decreases, which is generally associated with female attraction [43]. Our results confirm that both, the CS and the EX approach are suited to documenting the song behaviour of the nightingale over the course of its breeding season. We hence propose that CS recordings are a valuable addition to conventional nightingale studies and this approach should also be applied to studies on other vocal bird and animal species.

EX recordings had a higher percentage of data that was valid for further analysis, yet CS data had a higher total number and total duration of valid nightingale song recordings. Despite the given limitation in a maximum recording duration of two minutes, the cumulated recording time of the CS data was significantly higher than the EX data. This highlights that also with many short recordings a large dataset may be generated. The required recording length certainly depends on the specific research questions. For example, long recordings may be needed for song analyses on an individual level and also depend on the species’ song repertoire, i.e. the number of song types. Based on our definition, non-identifiable song type recordings have a reasonable recording quality that could not, however, be used for dialect research since complete song types without any missing elements should be considered for analysis. But they might however be useful for further structural analyses, e.g. based on elements and syllables. Citizens have also recorded other bird sounds that were no nightingales. On the one hand, a song very similar to the nightingale could have caused these false identifications, on the other hand, many citizen scientists may have suspected that only nightingales sing at night. However, due to light pollution in urban areas, species such as the blackbird, *Turdus merula*, and the robin, *Erithacus rubecula*, also sing at night [65, 66]. During the quality check of all recordings, we noticed that citizen scientists often only tested the ‘Naturblick’ app without the intention to record a nightingale at this particular moment. On other occasions, nightingales may have ceased singing, when the recording started. These circumstances, among others, might have led to ‘no bird‘ recordings.

The citizen scientists in our case study created a large dataset without any form of an external incentive to take extensive recordings of nightingales (e.g. badges or other award systems). The widespread notion that there is a lack of intrinsic motivation in participants without an external reward system [21, 22], was in our case not true. Citizen scientists generated many ‘ist’ recordings even without video instruction or assistance from a scientist and lack of knowledge and skills. Yet, with an instructional video and a detailed explanation of which kind of recordings should be generated and why, the number of recordings possibly would have been even higher in number, mean duration and/or quality. For dialect research, many recordings with many song types from different males as well as from different regions is more helpful than a few recordings of selected individuals at one or few location(s). Previous research has demonstrated that the repertoire of a nightingale male is well represented with one-hour recordings [e.g. 64]. Long-term stationary recordings of an individual would be, for example, advantageous when studying changes in song behaviour in the course of the breeding season. However, large numbers of geographically widespread citizen scientists offer novel potential for dialect research. For instance, different populations with a large distribution range can be investigated in a short period of time, which a single research group would not be able to achieve. Our study underlines that citizen scientists facilitate large-scale data collection. Furthermore, our results are in contrast to the notion that special knowledge is crucial to generate a large valid dataset [21, 22]. Additionally, our results contradict the assumption that it is always necessary to instruct citizen scientists by scientists to obtain better data [58]. At least not for the investigation of nightingale song dialects that would be based on visual inspection of spectrograms; the bioacoustic research question that we chose as background for a criterion for data quality.

Our comparison among the three user groups within the CS project (single, frequent and power users) showed that as predicted, user groups generated a dataset of different recording quality. We demonstrated that with the higher number of recordings in the group of power users, the percentage of recordings with valid quality for further analysis was also higher—without any special instructions or training by scientists. Furthermore, ‘no bird’ song recordings made up the lowest percentage in all user groups followed by other bird species recordings. The latter indicates that with the support of the pattern-recognition of the ‘Naturblick’ app, all user types were equally good at distinguishing nightingale from vocalizations of other bird species. Although the power users generated the most high-quality nightingale recordings, the larger group of frequent users in terms of the number of participants contributed recordings with the longest cumulative recording time and the largest cumulative number of song types. We conclude that even without extensive training, citizen scientists are able to generate recordings of valid quality. The assumption that citizen scientists must be trained and instructed over a longer period of time [11] was hence not true for this case study. We believe that in the science of citizen science [67] a general assessment of the data quality, training needs as well as required knowledge and skills of participants is not possible. We feel that it is particularly important to consider the skill and knowledge requirements specific to the research questions when planning CS activities.

### The technical influence on CS data

Several differences that we found between CS and EX recordings are most likely not due to training deficits in citizens, but down to technical differences in the recording devices used. Experts had a higher relative percentage of valid recordings determined via SNRs than citizen scientists. We assume, however, that this was not exclusively caused by the fact that the citizen scientists produced less and poorer nightingale recordings with an unqualified recording behaviour, but was also due to the technical limitations of some smartphone brands. For example, studies also showed that microphones with a low SNR lead to noisy recordings in which weak and/or distant signals are no longer to be identified clearly [68]. Darras and colleagues [69] showed that even using different professional microphones resulted in quite different SNRs. Thus, the quality of audio recordings is not only influenced by training but mostly by the choice of device i.e. microphone quality. Some of the EX recordings in our data generated with professional equipment were yet marked via the SNR values as poor in quality. This underlines that low recording quality is not a phenomenon that can and should be attributed exclusively to CS data.

We found lower SNRs in CS recordings for all song categories than in EX recordings because smartphones have limitations in frequency. Smartphone microphones are optimized for the frequency range of human speech (300–5,000 kHz), and their directional characteristic suppresses surrounding noise, especially in the bass range [70]. An earlier study showed similar results to ours: Smartphones only generated reliable recordings in a range of 300–3,400 Hz and uncontrollable compression levels occurred at higher frequencies [71]; in fact, exactly the range of maximum frequencies (up to 10 kHz) found in the nightingale’s song. Furthermore, Yousefian and Loizou [72] demonstrated that some smartphones use several microphones to separate the ambient noise from the speech during phone calls, and Martın-Donas and colleagues [73] found that the more microphones a smartphone has, the more background noise it filters out. This kind of audio pre-processing is further perturbing the frequency sensitivity of the recording devices used by CS. Moreover, most of the EX recordings were generated with the PMD and an external Sennheiser microphone.

We expected that due to their long-range transmission characteristics [44], SNRs of whistles would be better than the SNRs of trills, since their signal strength decreases faster over distance [45, 46]. We detected this in the EX recordings, but not in the CS recordings. In the former, the SNR values of the whistles and trills were equal (12 dB). In the latter, the SNR values of the whistlers were higher (19 dB) than those of the trills (16 dB). Furthermore, in the EX recordings, the SNRs of the buzz were 18 dB higher than the trills and almost as good as the whistles. However, the song category of the buzz had the lowest SNRs (below 10 dB) in CS recordings and was therefore by definition of [56] not of valid quality. However, referring to other sources such as [51, 59], which define valid quality recordings above at an SNR of 5 dB, all CS recordings would be of valid quality whereby e.g. durations can be measured. Thus, depending on how strict the threshold is, either only the whistles and trills (at 10 dB) or also the buzz songs (at 5 dB) are of valid quality. In the nightingale, buzz song types are an indicator of the quality of a male [50] and may therefore be presented at high pitched volume. Trill song types are used in aggressive interactions [74] and as an indicator of male quality [75], which may have led to a greater range and thus to better SNRs in general. Nevertheless, all CS recordings showed a significant lower SNR value than the EX recordings. Thus, the CS recordings were not equal to EX recordings, but still of valid quality. From personal experience, we can say that the significantly worse SNRs of CS recordings did not, however, lead to the fact that song types could be assigned to categories or types more poorly. Therefore, we believe that our CS recordings of nightingales can be used for further research questions, such as dialect research since the study of regional variations in bird song is usually based on the relative occurrence of song types [75], rather than spectral parameters.

In our test recordings, quite large deviations from the original values were found in the parameters minimum and maximum frequency as well as for song durations. The device producing the least deviation from the original’s minimum and maximum frequencies in its recordings was the professional PMD. The Zoom H2n, which is also used as professional equipment however, showed the greatest deviation from the original recording at both the minimum and maximum frequency. This shows that even a professional recorder without an external microphone may provide even worse measurement values than smartphones. One reason for this could be that the Zoom recorder is in particular designed to be used for long-term monitoring recordings and not for fine structure analyses. This is because long-term monitoring surveys use pattern recognition algorithms to determine the potential occurrence of bird species by analyzing vocalizations. Here mainly frequency and duration ranges are used instead of precise measurements, which also allows the use of recordings with lower SNRs [76]. This is in line with our data, which showed that the SNR values of the Zoom recorder were valid for our further analyses. Out of the smartphones, the recordings of the HTC (a low-cost brand) had very large deviations from the acoustic parameters of the original recordings. The duration most likely showed large deviations, because not all frequencies were recorded and thus the song type was not represented in its entirety with all elements and syllables. The smartphone brands Apple, Nexus and Samsung showed a significantly larger deviation in the frequencies than the PMD, but were comparable in the durations. Interestingly, in terms of song duration, the Samsung smartphone devices performed better than the PMD. Our test in comparing the recording quality of the devices showed that the quality of the brand, and thus ultimately the price, actually played an important role here in the frequency measurements, but not in the measurement of durations. Hence, the statement that the use of different recording devices can be neglected in the analysis of nightingale songs because of their very stereotypical song learning [49], does not apply when comparing measurements of frequencies of recordings that were made with different recording devices. Clare and colleagues [77] already described that measurements alone cannot accurately determine the effectiveness and usability of a dataset. The authors recommended that data quality should be presented as a kind of threshold value, which is derived from both data accuracy and the intended analyses. They suggested that how data quality is assessed, indeed depends on the research question. The question investigated here was whether CS recordings of nightingale may be used for song dialect research. Our prediction that the quality would be first, valid for this research question and second, comparable to EX data, could by large be confirmed.

## Conclusion and recommendations

Our study shows that nightingale recordings generated via a smartphone app are valid to investigate dialects at the song type level. Based on our results of the poor recording quality of low-cost smartphone brands, we would recommend the use of external and regularly calibrated microphones for projects relying on the analysis of fine structures. Kardous and colleagues [78] already recommended the utilization of external, calibrated microphones to improve the overall accuracy and precision of sound recordings. They showed that this eliminated much of the variability and limitations of built-in smartphone microphones. Furthermore, when measuring frequencies and durations, we suggest to ideally always use the same brand of recording devices, so that any differences found are due to song variations and not to discrepancies in the microphone used. Standardizations with regard to citizen scientists’ devices, e.g. by equipping them through the project or recommending the use of certain brands, could provide a solution for small projects and without continental-scale.

Despite the limited academic experience of citizen scientists, we strongly advocate that CS can make valuable contributions to science itself. In view of our results, we believe that CS recordings offer the potential to support bioacoustic and in particular dialect research with extensive datasets. Our case study demonstrated that dialect research on the song type level of the nightingale can be carried out with both CS and EX recordings. We support the notion of Butcher and Niven [17] as well as Lisjak and colleagues [18], stating that CS may complement and potentially replace conventional data sources. Based on our findings, we thus want to encourage bioacoustic researchers to first, use data made available by volunteers and non-academics, such as the recordings in open databases like XenoCanto (https://www.xeno-canto.org/) for instance in dialect research and second, to further establish CS as a research approach that has the dual benefit of providing large, and with newer technology also valid data, as well as opening science to society.

## Supporting information

S1 Table Comparison of functional types of recordings by CS recordings with the ’Naturblick’ app and six EX recordings with traditional microphones in the years 2018 and 2019. The representation of the raw data are given as subsumed numbers. Comparison between the relative percentage of recordings with valid quality for further analysis between CS and EX data. https://doi.org/10.5281/zenodo.4817236.
(PDF)

S2 Table Comparison of functional types of recordings in the years 2019, based on 245 one-time users, 361 frequent users and 18 power users recordings with the ’Naturblick’ app. One-time users have generated one recording, multiple users shared on average 2–19 recordings and frequent users shared on average over 20 recordings. Comparison between the relative percentage of recordings with valid quality for further analysis between CS data among different user types. https://doi.org/10.5281/zenodo.4817236.
(PDF)

S3 Table Comparison of the deviation of acoustic parameters for the minimum frequency, the maximum frequency and song duration from a sample of nine nightingale song types between citizen science recordings (CS) using different smartphone brands and expert recordings (EX) using equipment with professional microphones. Comparison between the playback test recordings of CS and EX recording devices (smartphones vs. professional equipment).
(PDF)
