# Supplementary material for: Opportunities and limitations: A comparative analysis of citizen science and expert recordings for bioacoustic research
Source: PLoS One. 2021 Jun 28;16(6):e0253763. doi: 10.1371/journal.pone.0253763 (PMC8238189; doi:10.1371/journal.pone.0253763)
Supplement: S1 Table — The representation of the raw data are given as subsumed numbers. Comparison between the relative percentage of recordings with valid quality for further analysis between CS and EX data. https://doi.org/10.5281/zenodo.4817236. (PDF) [file pone.0253763.s001.pdf]

	2018		2019	
	CS	EX	CS	EX
Number of identifiable song type recordings	1087	6	3036	6
Number of non-identifiable song type recordings	740	0	2111	0
Number of nightingale call recordings	6	0	82	0
Number of recordings of other bird species	427	0	214	0
Number of no birds recordings	165	0	236	0
Mean duration of recordings (s)	58	3600	54	3600
Cumulative recording time of all recordings (h)	39	6	89	6
