# Supplementary material for: Opportunities and limitations: A comparative analysis of citizen science and expert recordings for bioacoustic research
Source: PLoS One. 2021 Jun 28;16(6):e0253763. doi: 10.1371/journal.pone.0253763 (PMC8238189; doi:10.1371/journal.pone.0253763)
Supplement: S2 Table — One-time users have generated one recording, multiple users shared on average 2–19 recordings and frequent users shared on average over 20 recordings. Comparison between the relative percentage of recordings with valid quality for further analysis between CS data among different user types. https://doi.org/10.5281/zenodo.4817236. (PDF) [file pone.0253763.s002.pdf]

	One-time users	Frequent users	Power users
Number of identifiable song type recordings	104	708	373
Number of non-identifiable song type recordings	88	644	57
Number of nightingale call recordings	5	35	3
Number of recordings of other bird species	21	44	2
Number of no birds recordings	6	26	7
Mean duration of recordings (s)	51	62	85
Cumulative recording time of all recordings (h)	4	34	8
