# Supplementary material for: Opportunities and limitations: A comparative analysis of citizen science and expert recordings for bioacoustic research
Source: PLoS One. 2021 Jun 28;16(6):e0253763. doi: 10.1371/journal.pone.0253763 (PMC8238189; doi:10.1371/journal.pone.0253763)
Supplement: S3 Table — Comparison between the playback test recordings of CS and EX recording devices (smartphones vs. professional equipment). (PDF) [file pone.0253763.s003.pdf]

	CS										EX	
	Nexus 5x (2015)		HTC One M8s (2015)		iPhone 6 (2015)		iPhone 7 (2016)	Samsung Galaxy J3 (2016)	Samsung Galaxy S7 (2016)	Samsung Galaxy S10 (2019)	PMD660	Zoom H2n
Mean minimum frequency whistle 01	1470	1630	1815	1730	1580	1670	1760	1645	1555	1600	1560	1610
Mean maximum frequency whistle 01	8695	8725	6595	7620	8660	8670	8590	6780	10440	8685	10545	8520
Mean duration frequency whistle 01	2.4875	2.5555	2.4725	2.258	2.2565	2.496	2.465	2.531	2.5015	2.486	2.487	2.195
Mean minimum frequency whistle 02	1550	1665	1675	1740	1660	1670	1740	1385	1490	1515	1135	1610
Mean maximum frequency whistle 02	8070	8330	7285	7570	10155	8310	7730	6930	8325	8415	8070	7470
Mean duration frequency whistle 02	3.4155	3.44	3.361	3.4005	3.3695	3.346	3.379	3.4005	3.414	3.405	3.4045	3.362
Mean minimum frequency whistle 03	1635	1660	1610	1750	1815	1700	1670	1695	1640	1590	1565	1780
Mean maximum frequency whistle 03	7910	1660	8305	7565	8920	10370	10310	7425	4.425	7670	10340	7360
Mean duration frequency whistle 03	4.415	1660	4.425	4.4265	4.4495	4.429	4.444	4.4305	10410	4.4395	4.4085	4.409
Mean minimum frequency buzz 01	1300	1660	1515	1200	1365	1240	1350	1235	1295	1300	1200	1290
Mean maximum frequency buzz 01	7490	1660	7360	6605	7630	7360	7600	7090	7825	7445	7485	7340
Mean duration frequency buzz 01	1.7575	1660	1.7425	2.065	1.768	1.756	1.692	1.7665	1.769	1.7765	1.717	1.751
Mean minimum frequency buzz 02	1750	1660	1635	1675	1730	1700	1520	1760	1685	1600	1415	1630
Mean maximum frequency buzz 02	8070	1660	4790	7390	7340	10680	7380	7100	10590	7585	10730	7210
Mean duration frequency buzz 02	3.0305	1660	1.604	3.0415	3.0375	2.998	3.013	3.0495	3.0295	3.033	3.004	3.01
Mean minimum frequency buzz 03	1245	1660	1460	1555	1340	1290	1310	1155	1310	1520	1180	1440
Mean maximum frequency buzz 03	7930	1660	6895	6810	7400	10550	10420	6905	10425	7415	10720	7680
Mean duration frequency buzz 03	3.595	1660	3.628	3.602	3.6175	3.573	3.585	3.6335	3.6075	3.615	3.584	3.567
Mean minimum frequency trill 01	1545	1660	1480	1545	1640	1630	1550	1500	1530	1580	1255	1590
Mean maximum frequency trill 01	7650	1660	7530	7470	7500	7530	7530	7380	7670	7680	8790	7550
Mean duration frequency trill 01	2.022	1660	2.0355	2.087	2.062	2.055	1.997	2.065	2.0715	2.077	1.9665	2
Mean minimum frequency trill 02	1200	1660	1535	1440	1200	1290	1420	1050	1545	1230	1170	1390
Mean maximum frequency trill 02	7810	1660	5920	6455	7835	7790	7900	6325	7835	7735	8695	7340
Mean duration frequency trill 02	2.0435	1660	2.026	2.0785	2.026	2.023	2.018	2.0705	2.03	2.006	1.9925	2.066
Mean minimum frequency trill 03	1570	1660	1415	1460	1460	1590	1650	1245	1555	1380	1285	1700
Mean maximum frequency trill 03	8360	1660	7615	7930	8210	10570	10370	8095	10525	8090	10580	7550
Mean duration frequency trill 03	3.3525	1660	3.3595	3.3855	3.3685	4.058	4.07	3.3335	4.081	4.078	4.065	4.06
